# Supplementary material for: Splice-Junction-Based Mapping of Alternative Isoforms in the Human Proteome
Source: Cell Rep. Author manuscript; Available in PMC 2020 Jan 15. (PMC6961840; doi:10.1016/j.celrep.2019.11.026)
Supplement: 3 [file NIHMS1546469-supplement-3.zip › DF2/PXD006675/PulmonaryValve-97-Q14315-FGGEHIPNSPFHVLATEEPVVPVEPMES.pdf]

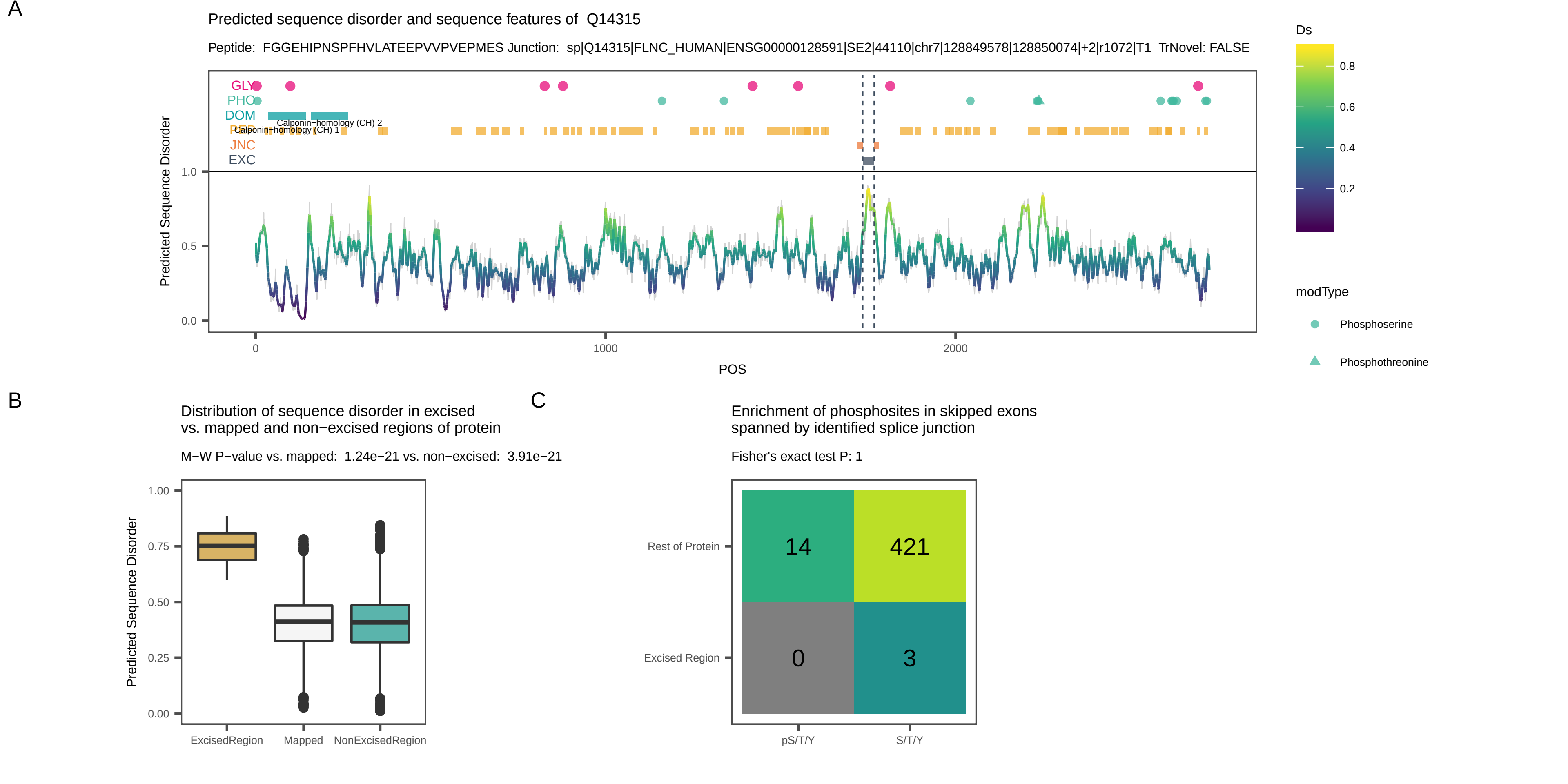
A
Predicted sequence disorder and sequence features of Q14315
Peptide: FGGEHIPNSPFHVLATEEPVVPVEPMES Junction: sp|Q14315|FLNC_HUMAN|ENSG00000128591|SE2|44110|chr7|128849578|128850074|+2|r1072|T1 TrNovel: FALSE
GLY
PHO
DOM
PEP
JNC
EXC
Calponin−homology (CH) 2
Calponin−homology (CH) 1
Predicted Sequence Disorder
1.0
0.5
0.0
0
1000
2000
POS
Ds
0.8
0.6
0.4
0.2
modType
Phosphoserine
Phosphothreonine
B
Distribution of sequence disorder in excised
vs. mapped and non−excised regions of protein
M−W P−value vs. mapped: 1.24e−21 vs. non−excised: 3.91e−21
Predicted Sequence Disorder
1.00
0.75
0.50
0.25
0.00
ExcisedRegion
Mapped
NonExcisedRegion
C
Enrichment of phosphosites in skipped exons
spanned by identified splice junction
Fisher's exact test P: 1
Rest of Protein
Excised Region
14
421
0
3
pS/T/Y
S/T/Y
